# Transfer-Matrix Framework for Modeling Mid-Infrared Vibrational Circular Dichroism Spectra

**DOI:** 10.1021/acs.analchem.5c07726

**Published:** 2026-03-24

**Authors:** Anton Utyushev, Ilia L. Rasskazov, Yamuna Phal

**Affiliations:** † Department of Electrical Engineering, 3557 Colorado School of Mines, 1610 Illinois St, Golden, Colorado 80401, United States; ‡ Independent Researcher, San Jose, California 95124, United States; § Quantitative Biosciences and Engineering Program, Colorado School of Mines, Golden, Colorado 80401, United States; ∥ Quantum Engineering Program, Colorado School of Mines, Golden, Colorado 80401, United States; ⊥ Colorado Clinical & Translational Sciences Institute (CCTSI), Aurora, Colorado 80045, United States

## Abstract

We present a rigorous electromagnetics-based theoretical model for mid-infrared (mid-IR) absorption and vibrational circular dichroism (VCD) spectra of homogeneous samples. The formalism based on 4 × 4 transfer-matrix methodology enables quantitative interpretation of the VCD signal for samples of varying thicknesses and under different incidence angles. Using an idealized material with distinct absorption bands, we explicitly show how the interference effects and coupling between left- and right-circularly polarized (LCP and RCP) waves contribute to the measured VCD signal. Importantly, contrary to conventional wisdom in mid-IR VCD, we show that tightly focused illumination can suppress the coupling between the LCP and RCP, which leads to a cleaner and more interpretable VCD signal. Our findings provide theoretical guidance for experimentalists to identify regimes where reliable mid-IR VCD measurements can be obtained and to recognize conditions dominated by optical artifacts from interference and coupling between LCP and RCP waves.

## Graphical abstract

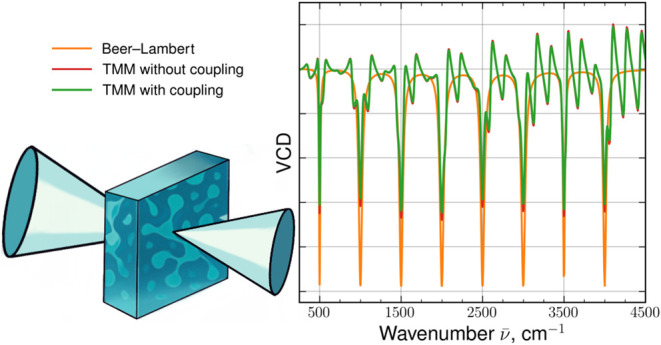

## Introduction

Chirality plays a central role in molecular recognition, catalysis, and biology. Detecting and quantifying the handedness through light–matter interactions has driven the development of chiroptical spectroscopy, where differential absorption of circularly polarized light reveals structural information. Among these techniques, vibrational circular dichroism (VCD) uniquely probes molecular vibrations in the mid-infrared, providing chemically specific insights into stereochemistry and conformational structure. The theoretical foundation of VCD is well established through quantum-mechanical and density-functional methods that relate vibrational transitions to molecular handedness.^1–3^ Experimentally, the first Fourier-transform infrared (FT-IR) VCD measurements^4^ led to decades of advances in instrumentation and data interpretation.^5–8^

Despite this progress, practical VCD measurements remain highly susceptible to optical artifacts. Real samples rarely obey the idealized assumptions underlying the Beer–Lambert law: thin films, supported layers, and partially oriented biomolecular samples can produce baseline offsets and apparent chiroptical signals originating from leakage of linear dichroism or birefringence.^9–12^ Recommended limits on path lengths and film thicknesses exist to reduce such artifacts,^13,14^ yet even under controlled conditions, false VCD remains a recurring issue. Similar complications are well documented in other chiroptical modalities, including photothermal circular dichroism and Raman optical activity.^15–17^

In recent years, quantum cascade laser (QCL) technology has enabled high-brightness, rapidly tunable mid-IR sources, making VCD instrumentation faster, more compact, and more suitable for imaging.^18–22^ In parallel, advances in chiral photonic materials have extended the concept of optical chirality into plasmonic, dielectric, and cavity-based platforms.^23–30^ However, a quantitative model that connects intrinsic chiral parameters to the VCD signal under realistic optical conditions remains missing. The transfer-matrix method (TMM) provides a rigorous route toward such a model. Originally formulated for stratified anisotropic media^31,32^ and later extended to chiral systems,^33,34^ the TMM captures the mutual coupling of left- and right-circularly polarized waves. Recent works^35,36^ established a general theoretical framework for linear chiral multilayers but did not systematically examine mid-IR VCD.

In this work, we develop a rigorous theoretical framework that explicitly incorporates circular-polarization coupling, multilayer interference, and illumination via focusing optics in mid-IR VCD. We frame TMM to gain direct insights into how interference and the coupling between LCP and RCP waves contribute to observable VCD spectra, all within the following key assumptions: (i) illumination is modeled as an incoherent superposition of plane waves within a finite numerical aperture (NA) acceptance cone, corresponding to the focused-beam geometry used in modern mid-IR microspectroscopy and QCL-based VCD imaging systems; (ii) the sample is treated as a single, homogeneous, optically isotropic chiral layer with scalar permittivity ε­(*ν̅*) and chirality (Pasteur) parameter κ­(*ν̅*), with perfectly flat and parallel interfaces and negligible surface roughness. The current model is immediately applicable to (i) solid thin films or supported layers that exhibit no macroscopic linear birefringence (LB) or linear dichroism (LD) and have perfectly flat interfaces and (ii) liquid samples measured in cells with perfectly isotropic, strain-free windows (e.g., BaF_2_ or CaF_2_). Possible routes for the generalization of the presented theoretical formalism are addressed in the Discussion section.

## Methods

### Transfer-Matrix Method for Multilayered Stacks with Chiral Materials

We employ the transfer-matrix method (TMM), which provides an accurate and numerically stable framework for modeling the propagation of circularly polarized light in stratified chiral media. In what follows, we outline the extension of the classical 2 × 2 TMM, commonly used for isotropic media^37,38^ to the 4 × 4 formalism,^31,36^ which fully captures the coupling between left- and right-circularly polarized (LCP and RCP) waves within chiral layers.

Consider an interface between two linear, homogeneous, isotropic chiral media characterized by parameters (ε_1_, μ_1_, κ_1_) and (ε_2_, μ_2_, κ_2_), with electromagnetic impedances $\eta_{1}=\sqrt{\mu_{1}/\varepsilon_{1}}$ and $\eta_{2}=\sqrt{\mu_{2}/\varepsilon_{2}}$ , where ε is the dielectric permittivity, μ is the magnetic permeability, and κ is the chirality (Pasteur) parameter. Boundary conditions for the tangential components of the **E**, **H**, **D**, and **B** fields lead to a linear relation between the circularly polarized field amplitudes propagating forward (→) and backward (←) in each medium

$${\left(\begin{array}{l} E_{\to }^{+}\\ E_{\to }^{-}\\ E_{\leftarrow }^{+}\\ E_{\leftarrow }^{-} \end{array}\right)}_{1}=T{\left(\begin{array}{l} E_{\to }^{+}\\ E_{\to }^{-}\\ E_{\leftarrow }^{+}\\ E_{\leftarrow }^{-} \end{array}\right)}_{2}$$ 1

where the 4 × 4 transfer matrix **T** has the block form

2 $$T=\left(\begin{array}{ll} M_{T} & M_{R}\\ M_{R} & M_{T} \end{array}\right)$$

These relations provide the starting point for a self-consistent treatment of polarization mixing at each interface within a multilayered chiral structure. Within each homogeneous chiral medium *j* = 1, 2, the circularly polarized eigenmodes propagate with refractive indices $n_{j\pm }=\sqrt{\varepsilon_{j}}\pm \kappa_{j}$ and wave vectors *k*_*j*±_ = 2π*ν̅n*_*j*±_, where *ν̅* is the wavenumber in vacuum (reciprocal of the free-space wavelength). The angles θ_*j*±_ obey a generalized Snell’s law *n*_1±_  sin θ_1±_ = *n*_2±_ sin θ_2±_ for the two circular eigenstates.

The submatrices *M_T_* and *M_R_* are given as^36^

3 $$M_{T\left(R\right)}=\frac{1}{4}\left[\begin{array}{ll} \left(1+\frac{\eta_{1}}{\eta_{2}}\right)\left(1\pm \cos\theta_{2+}/\cos\theta_{1+}\right) & \left(\frac{\eta_{1}}{\eta_{2}}-1\right)\left(1\mp \cos\theta_{2-}/\cos\theta_{1+}\right)\\ (\frac{\eta_{1}}{\eta_{2}}-1)\left(1\mp \cos\theta_{2+}/\cos\theta_{1-}\right) & \left(1+\frac{\eta_{1}}{\eta_{2}}\right)\left(1\pm \cos\theta_{2-}/\cos\theta_{1-}\right) \end{array}\right]$$

where cos θ_2±_ = [1 – (*k*_1±_ /*k*_2±_)^2^ sin^2^ θ_1±_ ]^1/2^, *k*_*j*±_ = 2π*ν̅n*_*j*±_, and θ_1,2±_ are the incidence and refraction angles of the RCP and LCP components in each medium, respectively (see Figure 1a). The block structure of **T** makes it explicit how LCP and RCP components mix at the interface of the two media. By isolating the forward- and backward-propagating field components, we can obtain effective 2 × 2 transmission and reflection matrices that act on the forward-propagating amplitudes. For incidence from the left, this reduction is implemented by imposing the boundary condition (*E*_←_^+^, *E*_←_^–^)_2_ = (0, 0), which excludes incoming waves from the output side. Applying the boundary conditions to the block matrix in eq 3 yields the compact relations

4 $$\begin{array}{l} {\left(\begin{array}{l} E_{\to }^{+}\\ E_{\to }^{-} \end{array}\right)}_{2}=T_{\to }{\left(\begin{array}{l} E_{\to }^{+}\\ E_{\to }^{-} \end{array}\right)}_{1},,T_{\to }=M_{T}^{-1}\\ {\left(\begin{array}{l} E_{\leftarrow }^{+}\\ E_{\leftarrow }^{-} \end{array}\right)}_{1}=R_{\leftarrow }{\left(\begin{array}{l} E_{\to }^{+}\\ E_{\to }^{-} \end{array}\right)}_{1},,R_{\leftarrow }=M_{R}M_{T}^{-1} \end{array}$$

Under this condition, the transmitted field amplitudes for incidence from the left is given by $T_{\to }=M_{T}^{-1}$ . The above formalism explicitly includes polarization conversion between LCP and RCP components, a key effect in strongly chiral media or in the case of grazing-incidence conditions, as will be shown later.

**Figure 1:**
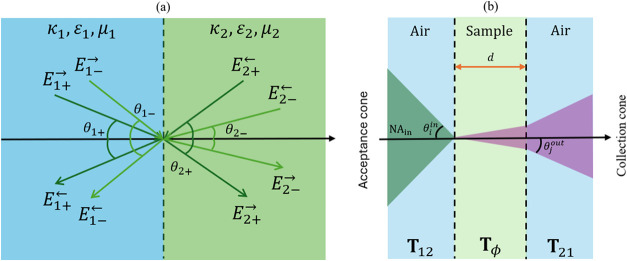
Schematic representation of electromagnetic-wave propagation (a) at the interface between two chiral media under plane-wave illumination and (b) in a multilayered stack under focused illumination with a given numerical aperture (NA). The illumination NA defines the set of incident angles θ_*i*_^in^ within the acceptance cone, while the detection NA selects the transmitted angles θ_*j*_^out^ within the collection cone. Only rays satisfying θ ∈ [0, min­(θ_max_^in^, θ_max_^out^)] contribute to the measured VCD signal.

In the case of a single chiral layer, the total transfer matrix is constructed by multiplying the interface matrices with the propagation matrix for that layer

5 $$T=T_{12}T_{\phi }T_{21}$$

where **T**_12_ and **T**_21_ describe transmission through the (1)–(2) and (2)–(1) interfaces, respectively (see Figure 1b), and **T**_ϕ_ accounts for propagation through the chiral layer of thickness *d*

6 $$T_{\phi }=\left[\begin{array}{llll} e^{-i\phi_{+}} & 0 & 0 & 0\\ 0 & e^{-i\phi_{-}} & 0 & 0\\ 0 & 0 & e^{i\phi_{+}} & 0\\ 0 & 0 & 0 & e^{i\phi_{-}} \end{array}\right],,\phi_{\pm }=k_{\pm }d=2\pi \mathrm{\nu ̅}n_{\pm }d$$

Here, *k*_±_ are complex wave vectors, so ϕ_±_ contain both phase accumulation and attenuation due to material absorption for the two circular eigenmodes. This matrix therefore captures both the phase accumulation and the absorption of LCP and RCP components throughout the layer.

The transmitted field amplitudes for incidence from the left are represented by the 2 × 2 complex transmission matrix

7 $$T_{\to }=\left[\begin{array}{ll} t_{++} & t_{+-}\\ t_{-+} & t_{--} \end{array}\right]$$

where each element *t*_*ij*_ connects the input circular polarization *j* to the output polarization *i*. The transmission matrix $T_{\to }$ is obtained directly from the total 4 × 4 transfer matrix of the system, eq 5, keeping in mind eq 2 and recalling that $T_{\to }=M_{T}^{-1}$ . We remind the Reader that $T_{\to }$ already includes transmission through both interfaces and the phase accumulation and absorption inside the chiral layer via the propagation matrix **T**_ϕ_ in eq 6. Specifically, *t*_++_ and *t*_––_ describe helicity-preserving transmission for right- and left-circularly polarized (RCP and LCP) light, respectively, while *t*_+–_ and *t*_–+_ account for polarization conversion between the two helicities.

The observable quantities in a typical VCD measurement are the transmitted intensities for incident RCP (+) and LCP (−) waves, obtained by summing the power in both preserved and converted components

8 $$T_{\pm }=\left|t_{\pm \pm }{|}^{2}+\right|t_{\pm \mp }{|}^{2}$$

This formalism provides a rigorous theoretical framework for modeling polarization-dependent transmission and reflection in chiral multilayer systems. Notice that at normal incidence, **T** becomes diagonal (see eq 3 with cos θ_1±_ = cos θ_2±_ = 1), so the cross-polarization terms vanish (*t*_+–_= *t*_–+_ = 0), and each helicity is transmitted independently.

In the achiral limit, κ → 0, the formalism is reduced to the classical TMM for isotropic thin films. The refractive indices collapse to a single value, *n*_+_ = *n*_–_ = $\sqrt{\varepsilon }$ , so that phases become identical, ϕ_+_ = ϕ_–_, and the interface matrices in eq 3 lose their off-diagonal structure. As a consequence, the 4 × 4 transfer matrix becomes block-diagonal, and no polarization conversion occurs: *t*_+–_ = *t*_–+_ = 0 for all angles. The transmitted intensities therefore satisfy *T*_+_ = *T*_–_, implying that all of the VCD measures vanish identically.

Having established eq 8 and defined the transmitted intensities of the right- and left-circularly polarized light, VCD is then defined as^35^

9 $$\mathrm{VCD}\left(\mathrm{\nu ̅}\right)=-\log_{10}\frac{T_{-}\left(\mathrm{\nu ̅}\right)}{T_{+}\left(\mathrm{\nu ̅}\right)}$$

Noteworthy, there are other alternative definitions of VCD used in the literature:^36^ VCD = Δ*T*/ *T̅*, where Δ*T* = *T*_+_ – *T*_–_ and *T̅* = (*T*_+_ + *T*_–_)/2. An algebraically equivalent form follows from the substitution *T* → *A*, yielding VCD = Δ*A*/ *A̅* with Δ*A* = *A*_+_ – *A*_–_ and *A̅* = (*A*_+_ + *A*_–_)/2.

### Dielectric Permittivity of an Ideal Absorber

For the sake of clarity of numerical results, we establish a hypothetical chiral material which is characterized by important feature: the absorbance spectrum of the respective thin film, calculated via the Beer–Lambert law in the achiral limit

10 $$A_{\mathrm{BL}}\left(d,\mathrm{\nu ̅}\right)=\frac{4\pi \mathrm{\nu ̅}n^{\prime \prime }\left(\mathrm{\nu ̅}\right)d}{\ln\left(10\right)}$$

is characterized by equidistant peaks with the same amplitude (Figure 2c). Here, *d* is the film thickness, and *n*″ is the imaginary part of the refractive index of an ideal absorber. The reason for modeling such a material is an easy and clear manifestation of any spectral artifact due to neatly established “ideal” absorptance (Figure 2c).

**Figure 2:**
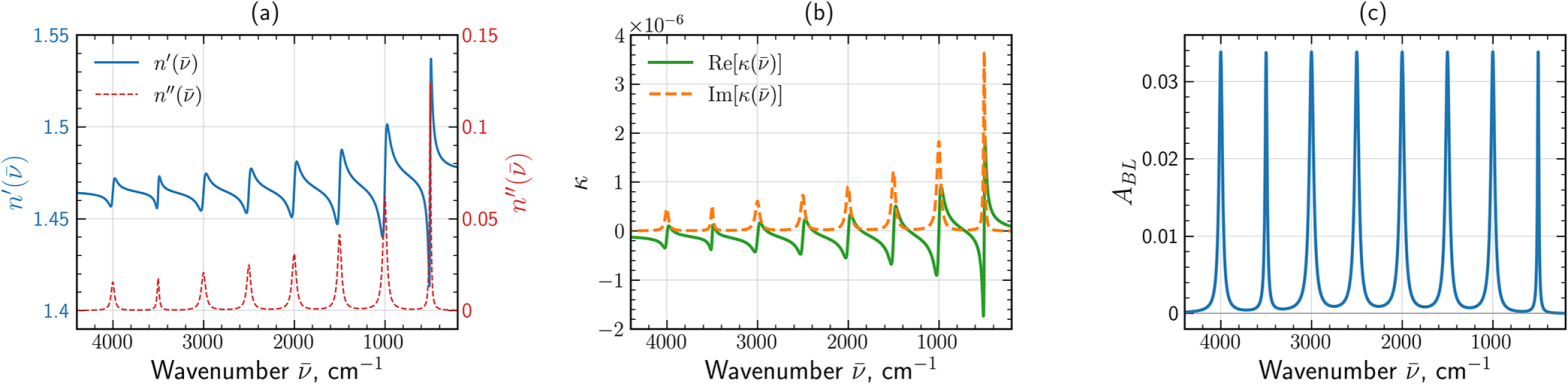
Optical properties of the ideal absorber: (a) real, *n*′(*ν̅*), and imaginary, *n*″(*ν̅*), parts of the refractive index; (b) real and imaginary parts of the chirality parameter κ­(*ν̅*); (c) Beer–Lambert absorbance, *A*_BL_, for *d* = 1 μm thick achiral sample with refractive index given in panel (a).

The complex dielectric function is modeled using a series of Lorentzian oscillators^36^

11 $$\varepsilon \left(\mathrm{\nu ̅}\right)=\varepsilon_{\infty }+\sum_{j}\frac{f_{j}{\mathrm{\nu ̅}}_{p}^{2}}{{\mathrm{\nu ̅}}_{0,j}^{2}-{\mathrm{\nu ̅}}^{2}-i\gamma_{j}\mathrm{\nu ̅}}$$

where ε_∞_ is the high-frequency permittivity, *ν̅* is the wavenumber, *f*_*j*_ is the oscillator strength, *ν̅*_0,*j*_ is the resonance wavenumber of the *j*-th mode, γ_*j*_ is its damping constant, and *ν̅*_*p*_ is an effective plasma wavenumber. The oscillator parameters are chosen so that the corresponding Beer–Lambert absorbance spectrum exhibits evenly spaced peaks of nearly equal amplitude (Figure 2).

Chirality is introduced through the Pasteur medium model, where the chiral admittance κ follows a similar Lorentzian formulation^36^

12 $$\kappa \left(\mathrm{\nu ̅}\right)=\kappa_{0}\sum_{j}\frac{f_{j}{\mathrm{\nu ̅}}_{p}^{2}\mathrm{\nu ̅}}{{\mathrm{\nu ̅}}_{0,j}\left({\mathrm{\nu ̅}}_{0,j}^{2}-{\mathrm{\nu ̅}}^{2}-i\gamma_{j}\mathrm{\nu ̅}\right)}$$

where *f*_*j*_ represents the strength of the chiral interaction.

Physically, the real part of κ corresponds to optical rotation (dispersive response), while the imaginary part governs differential absorption (circular dichroism). This decomposition highlights how microscopic chirality manifests in macroscopic observables that can be viewed via mid-IR VCD spectroscopy.

Figure 2a shows the calculated optical constants *n*′(*ν̅*), *n*″(*ν̅*), whereas Figure 2b shows real and imaginary parts of the chirality parameter κ­(*ν̅*). The complete set of oscillator parameters used to construct ε­(*ν̅*) and κ­(*ν̅*) is listed in Table 1.

**Table 1:** Parameters of the Equidistant Lorentzian Oscillators Used in the Dielectric and Chirality Function Model (see eqs 11 and 12)­^a^

*j*	*ν̅*_0,*j*_ (cm^–1^)	*f*_*j*_	γ_*j*_ (cm^–1^)
1	500	0.3000	26.91
2	1000	0.4119	37.17
3	1500	0.6374	57.56
4	2000	0.2882	26.18
5	2500	0.5695	51.47
6	3000	0.2043	18.51
7	3500	0.3678	33.28
8	4000	0.5413	48.91

a The high-frequency dielectric constant ε_∞_ = 2.15, the chirality constant κ_0_ = 10^–5^, and the effective plasma frequency *ν̅*_p_ = 107 cm^–1^ are constant for all oscillators.

It should be emphasized that the Beer–Lambert law provides the correct limit only for weak chirality and for normal incidence, where polarization mixing is negligible. In chiral thin films at oblique incidence, polarization conversion and angle-dependent phase accumulation break the assumptions underlying eq 10, so the Beer–Lambert prediction serves only as a baseline rather than as a quantitatively accurate model.

For large numerical apertures, and especially for long wavelengths where *n*_±_ < 1, the incidence could be near or above the critical angle and gives rise to strong phase variations and evanescent contributions in the transmitted fields. Importantly, in our idealized oscillator model, the real part of *n*_±_ may dip below unity near strong dispersion, so the argument of the arcsin function can exceed 1. This corresponds to evanescent contributions and phase singularities in the full 4 × 4 transfer-matrix solution and should not be taken as a representative of typical molecular films. These effects give rise to the oscillatory tails that appear in the VCD response near the band edges.

## Results

### Ideal Achiral Absorber

To establish a baseline for a subsequent analysis of chiral thin films, we first consider a hypothetical ideal *achiral* absorber described by the dielectric function (see Figure 2a), with the chirality parameter set to zero: κ = 0. We characterize the attenuation of the signal in mid-IR microspectroscopy by the absorbance

13 $$A\left(\mathrm{\nu ̅}\right)=-\log_{10}T\left(\mathrm{\nu ̅}\right)$$

where *T*(*ν̅*) is the transmittance.

Figure 3 compares the absorbance spectra computed using the transfer-matrix method and the idealized prediction via the Beer–Lambert law for film thicknesses ranging from 1 to 50 μm, all that for a single plane-wave illumination under normal incidence. For thin layers with *d* < 20 μm, the spectra calculated via TMM exhibit pronounced interference fringes arising from multiple reflections inside the slab, producing oscillatory deviations from the exponential decay predicted by the Beer–Lambert law. As the thickness increases, these fringes gradually diminish due to strong attenuation of the signal and the absorbance converges toward the seminal Beer–Lambert behavior.

**Figure 3:**
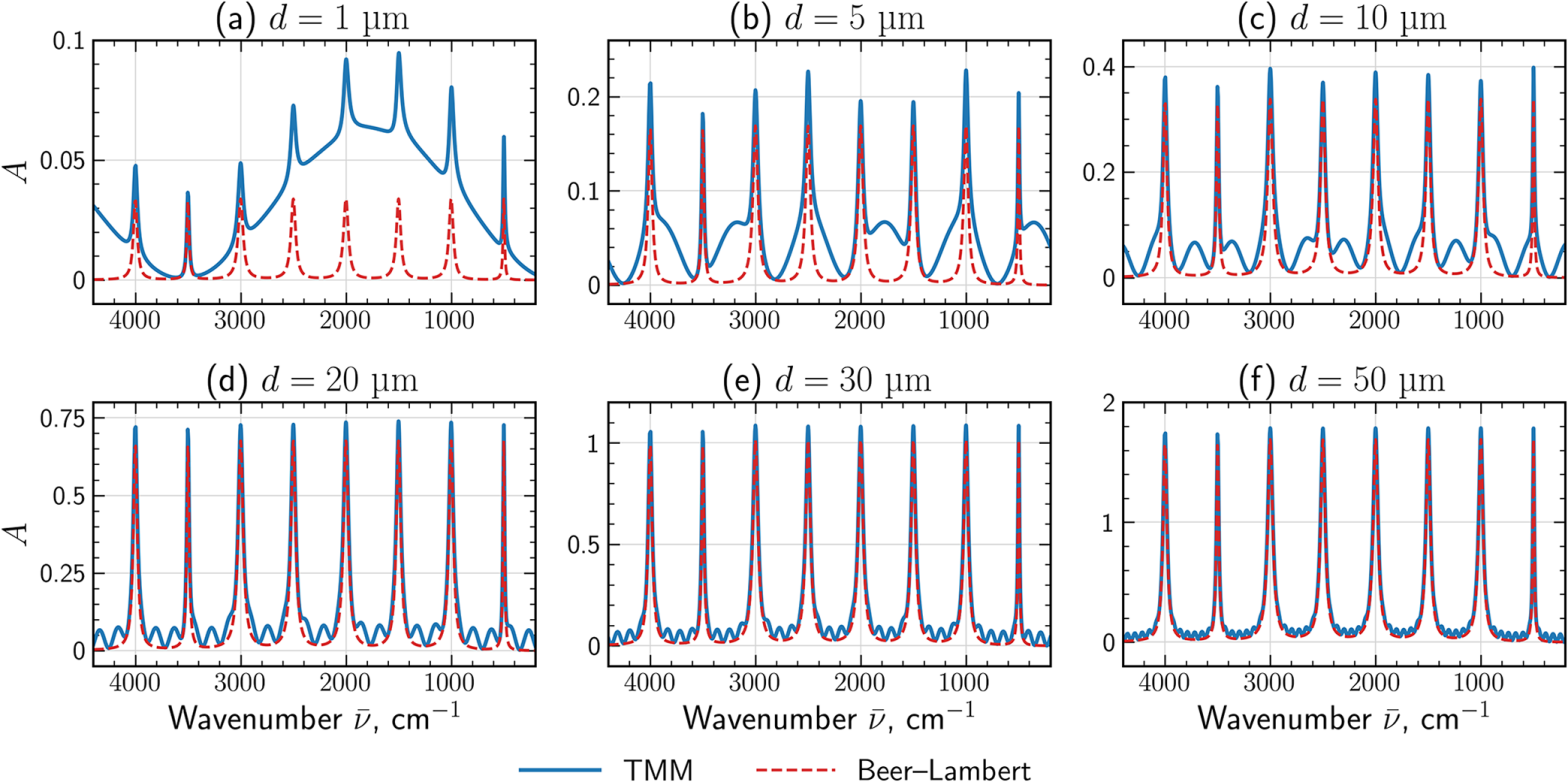
Absorbance spectra, *A*, of a hypothetical ideal achiral absorber (κ = 0), calculated with TMM (solid blue lines) and with the Beer–Lambert law (red dashed lines). Each panel corresponds to a different film thickness: (a) 1 μm, (b) 5 μm, (c) 10 μm, (d) 20 μm, (e) 30 μm, and (f) 50 μm.

The results (Figure 3) provide a fundamental reference to evaluate how a nonzero chiral admittance κ modifies light propagation. This baseline sets the stage for the next section, where the differential absorbance between the RCP and LCP polarizations, *A*_±_, generates the vibrational circular dichroism (VCD) signal defined in eq 9.

### Ideal Chiral Absorber

#### Normal Incidence

To investigate the emergence of the chiral optical response, we introduce a finite-frequency-dependent chirality parameter κ­(*ν̅*) defined by eq 12. This parameter leads to different propagation of right- and left-circularly polarized light, due to difference between refractive indices $n_{\pm }\left(\mathrm{\nu ̅}\right)=\sqrt{\varepsilon \left(\mathrm{\nu ̅}\right)}\pm \kappa \left(\mathrm{\nu ̅}\right)$ . The resulting asymmetry in absorption between the two helicities manifests as vibrational circular dichroism (VCD) calculated from eq 9.

Figure 4 shows the calculated absorbance spectra *A*_±_ and the corresponding VCD response for chiral films of thickness *d* = 1, 25, and 50 μm at normal incidence. Notice, *A*_±_ is calculated via eq 13, where respective *T*_±_(*ν̅*) has been used. For the thinnest film (*d* = 1 μm), the absorbances of the two circular polarizations are nearly identical, and the resulting VCD is extremely weak. At the same time, the interference effects are strongly pronounced. As the thickness increases, interference effects in *A*_±_ are reduced, but the overall dichroic signature is dominated by the absorptive part of the chiral parameter, Im­[κ­(*ν̅*)]. The negative VCD lobes coincide with the absorption resonances of Im­[κ], confirming that the dichroic signal stems from microscopic chiral absorption rather than a geometric interference artifact.

**Figure 4:**
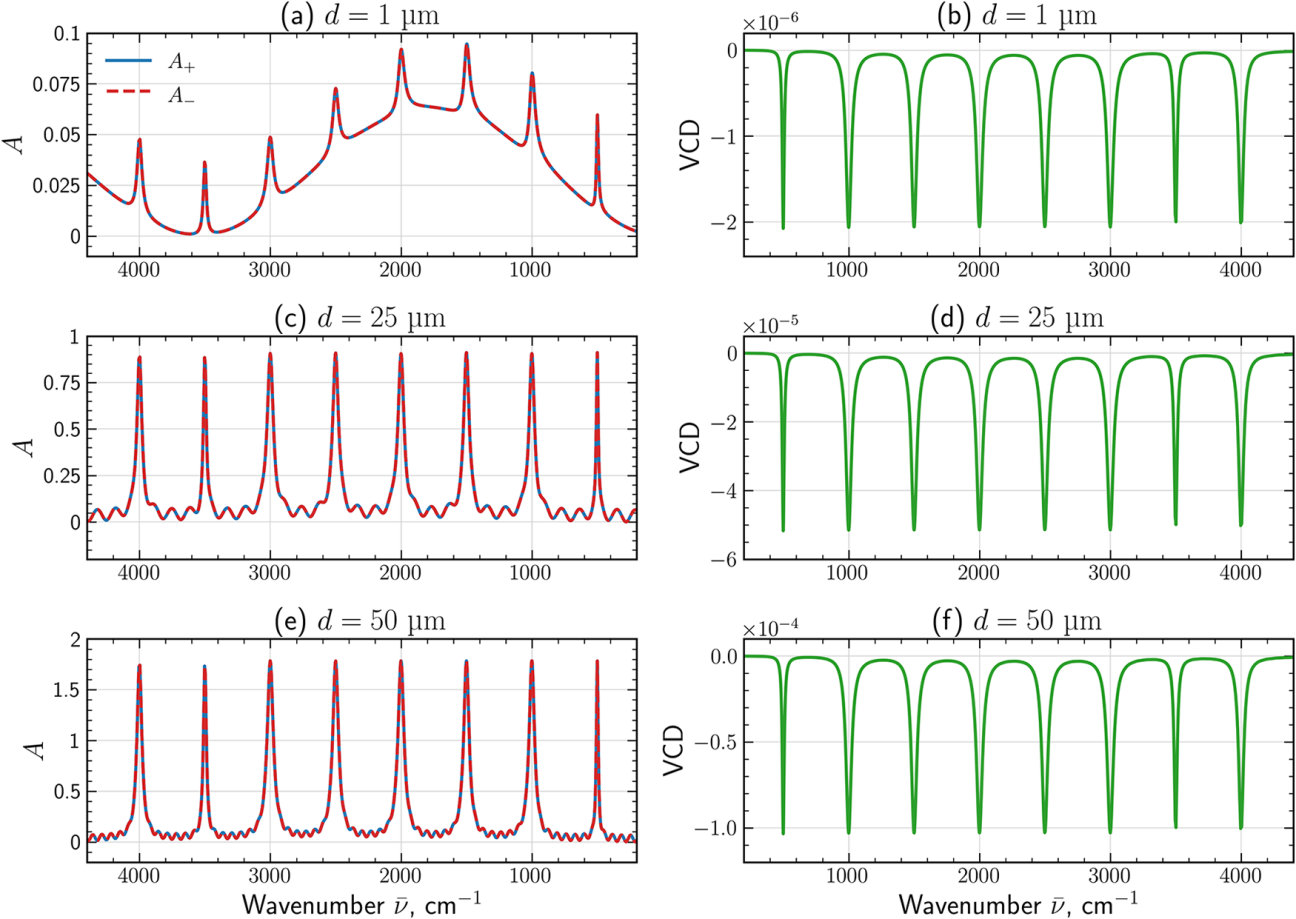
Absorbance *A*_±_ (left) and vibrational circular dichroism VCD (right) spectra of the ideal chiral absorber under normal plane-wave illumination for film thicknesses: (a, b) 1 μm, (c, d) 25 μm, and (e, f) 50 μm. VCD amplitude grows with increasing thickness, demonstrating the linear scaling of the chiral absorptive response with optical path length and Im­[κ­(*ν̅*)].

The magnitude of VCD increases with the optical path length: from ∼10^–6^ for a 1 μm film to ∼10^–4^ for a 50 μm film, placing the thicker samples within the detectable range of mid-IR VCD measurements.^35^ This behavior reflects the linear scaling of differential absorbance with both Im­[κ­(*ν̅*)] and the sample thickness.

Overall, these results establish a direct connection between the microscopic chiral oscillator model encoded in κ­(*ν̅*) and the macroscopic VCD spectrum. For weakly chiral media, the differential absorbance increases linearly with the magnitude of Im­[κ] and with film thickness, providing a consistent and quantitative basis for interpreting mid-IR VCD spectra of thin chiral layers.

#### Grazing Incidence

To examine how the angle of incidence affects the chiral optical response, we computed VCD spectra using eq 9 for layer thicknesses of *d* = 1, 10, and 25 μm under illumination with θ = 0°–80° (Figure 5(a–i)). We demonstrate the effects of interference and coupling between RCP and LCP waves by executing three sets of simulations for each case:

1. Rigorous solution via eq 9, which rigorously incorporates coupling effects between RCP and LCP waves, together with interference.
2. Same as above, yet mitigating the coupling between RCP and LCP waves. It is done by setting *t*_+–_ = *t*_–+_ = 0 in eq 8 and then using *T*_±_ = |*t*_±±_ |^2^ as an input for eq 9. Under this assumption, only interference effects are taken into account in the calculated VCD signal.
3. Using the Beer–Lambert law. The absorptances for RCP and LCP waves are calculated via eq 10, where respective *n*_±_^″^ are used. The optical thickness *d* → *d*/cos θ_*i*_^*in*^ is used for grazing incidence. Finally, eq 13 is used to get *T*_±_ from the relevant *A*_BL±_, and thus, to calculate VCD via eq 9.

The above controlled decomposition of the VCD signal allows to dissect interference and polarization coupling through progressively refined electromagnetic models.

**Figure 5:**
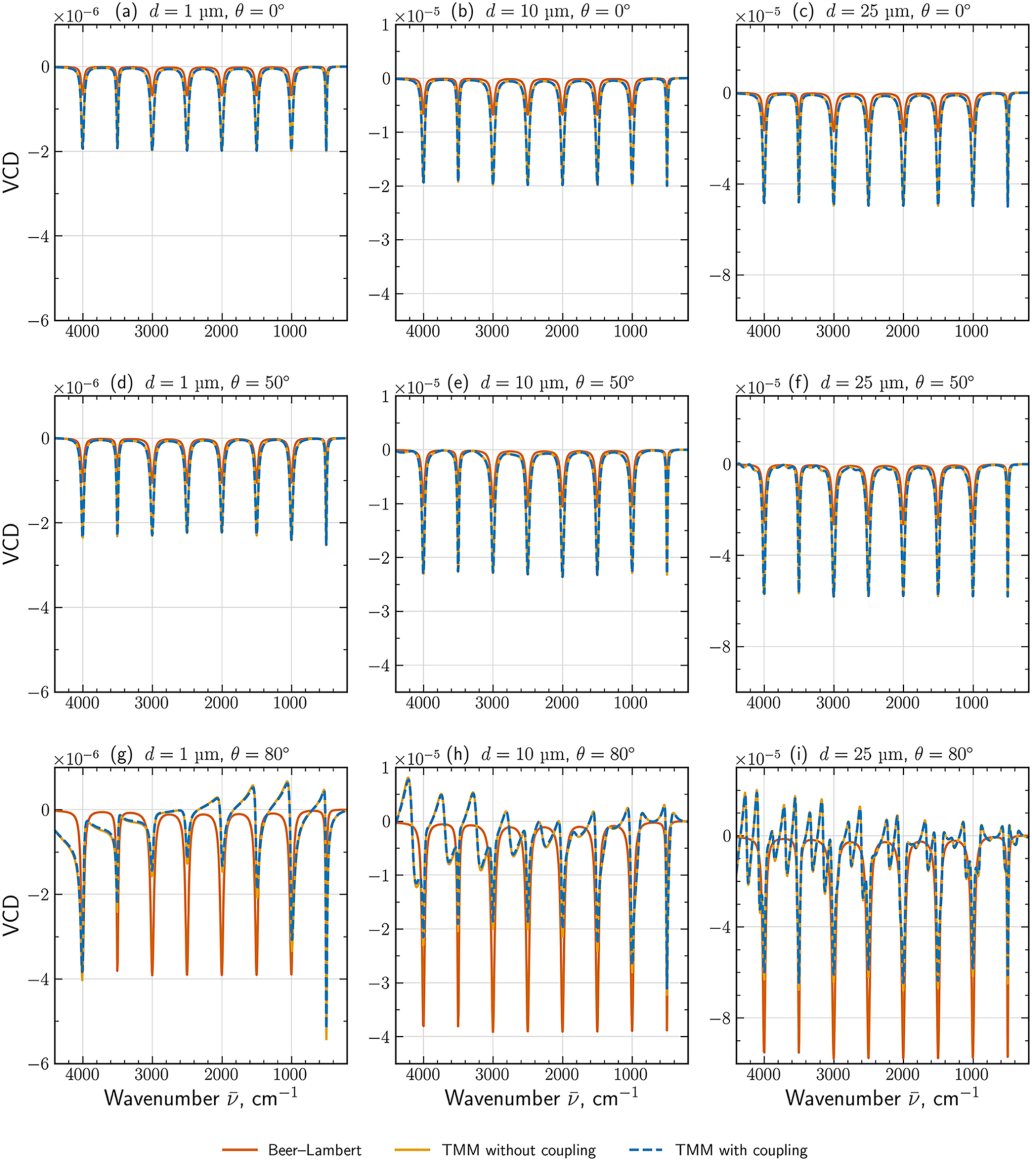
VCD spectra (a-i) calculated under different assumptions for chiral films with thicknesses of *d* = 1, 10, and 25 μm (columns) at incidence angles θ = 0°, 50°, and 80° (rows). Solid orange lines: Beer–Lambert model obtained with n±″=Im[ε±κ] and effective optical path *d*/cos θ (see eq 10). Solid yellow lines: VCD calculated via TMM, but taking into account only copolarized terms *T*_±±_ = |*t*_±±_|^2^, that is without considering LCP–RCP coupling. Dashed blue lines: VCD calculated via TMM taking into account total transmitted intensities *T*_±_ = |*t*_±±_|^2^ + |*t*_±∓_|^2^, that is, considering LCP–RCP coupling.

For a rigorous solution through TMM and eq 9 and for small angles of incidence, the spectra resemble the normal-incidence reference and scale predictably with film thickness, as can be seen from Figure 5(a–i). Larger incidence angles introduce interference and polarization-conversion effects that distort the spectral shape of the VCD, most pronounced in thicker films.

At oblique angles, small coupling effects appear as a result of polarization conversion and phase differences accumulated by the left- and right-circular components inside the film. This effect grows slightly with increasing thickness but remains modest across the studied range, confirming that the Beer–Lambert description captures only the intrinsic material response, whereas the transfer-matrix methods additionally include thin-film interference and polarization-coupling effects. To identify the relative contribution of interference and polarization coupling into the modeled VCD, we plot the following analysis in Figure 6. The first row in Figure 6(a–c) provides the reference VCD_BL_(θ, *ν̅*) calculated via the Beer–Lambert law. These maps represent the intrinsic material response expected in the absence of any other effects and therefore serve as a reference baseline for comparison with the rigorous transfer-matrix solution. The second row of Figure 6(d–f) isolates deviations arising from thin-film interference via

14 $$\Delta {\mathrm{VCD}}_{\mathrm{itf}}\left(\theta ,\mathrm{\nu ̅}\right)=\frac{{\mathrm{VCD}}_{\mathrm{BL}}\left(\theta ,\mathrm{\nu ̅}\right)-{\mathrm{VCD}}_{∥}\left(\theta ,\mathrm{\nu ̅}\right)}{{\mathrm{VCD}}_{\mathrm{BL}}\left(\theta ,\mathrm{\nu ̅}\right)}$$

where VCD_∥_ = −log_10_[*T*_–_/*T*_+_] is evaluated using only the copolarized transmission components *T*_±±_ = |*t*_±±_|^2^. This comparison preserves interference effects while suppressing helicity conversion, allowing the role of multiple internal reflections and angle-dependent phase accumulation to be quantified independently. The third row of Figure 6(g–i) isolates the additional contribution originating from LCP–RCP polarization coupling

15 $$\Delta {\mathrm{VCD}}_{\mathrm{cpl}}\left(\theta ,\mathrm{\nu ̅}\right)=\frac{\mathrm{VCD}\left(\theta ,\mathrm{\nu ̅}\right)-{\mathrm{VCD}}_{∥}\left(\theta ,\mathrm{\nu ̅}\right)}{{\mathrm{VCD}}_{\mathrm{BL}}\left(\theta ,\mathrm{\nu ̅}\right)}$$

where VCD = −log_10_[*T*_–_/*T*_+_] is evaluated with the total transmitted intensities *T*_±_ = |*t*_±±_ |^2^ + |*t*_±∓_|^2^ including both co- and cross-polarized contributions.

**Figure 6:**
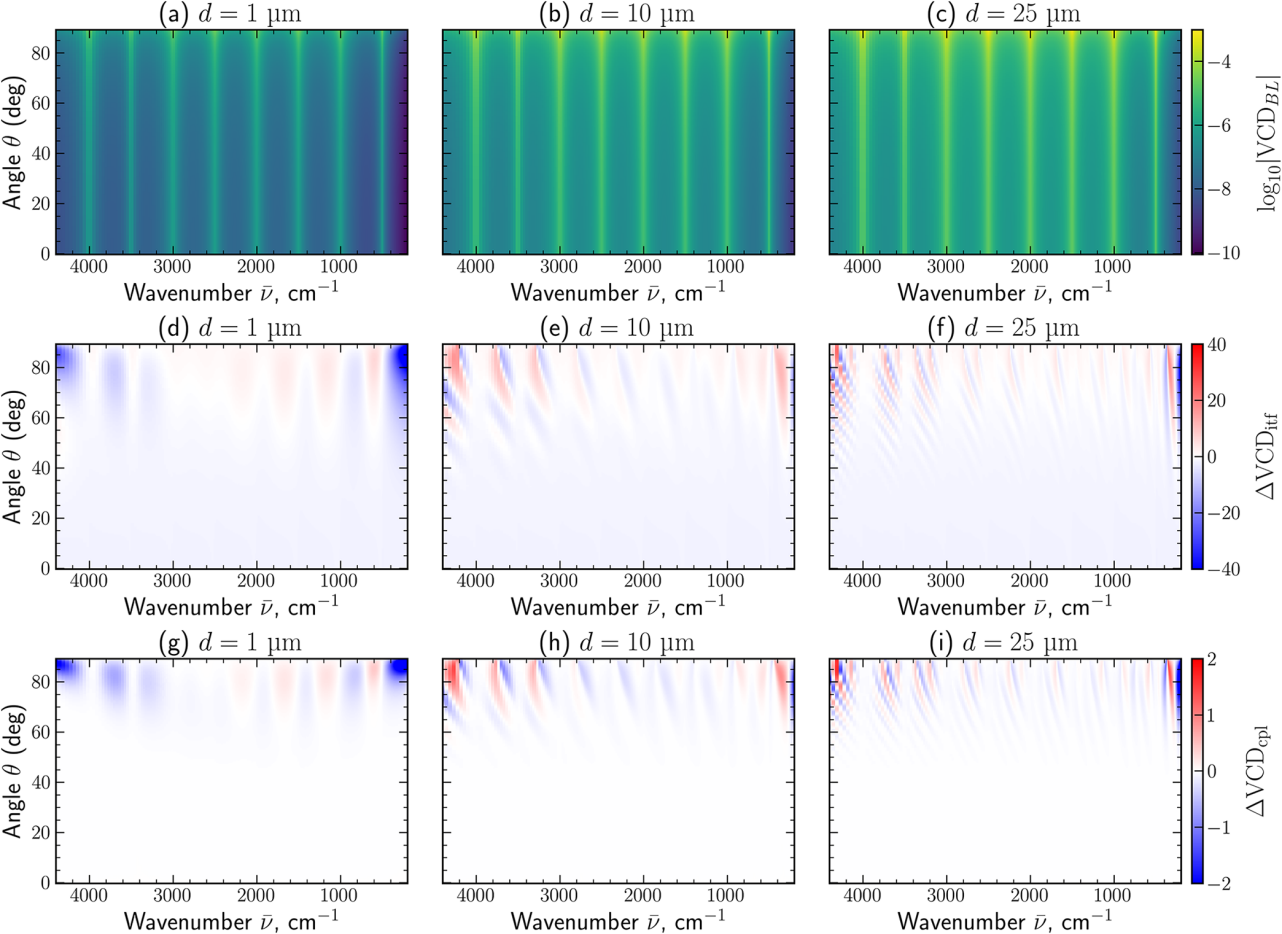
Angular–spectral maps for chiral films with thicknesses 1, 10, and 25 μm illustrating the separate electromagnetic contributions to the VCD signal. (a–c): log_10_|VCD_BL_| obtained from the Beer–Lambert law. (d–f): relative deviation, ΔVCD_itf_, isolating thin-film interference effects by comparison with the copolarized solution VCD_∥_. (g–i): relative deviation, ΔVCD_cpl_, quantifying additional changes caused by LCP–RCP polarization coupling in the full 4 × 4 transfer-matrix solution.

Figure 6 reveals a clear angular transition in the validity of simplified models. For small incidence angles (θ ≲ 60°), both deviations from the idealized Beer–Lambert law due to interference and cross-coupling remain close to zero, indicating a perfect regime for gathering clean VCD spectra. The strength and angular extent of these effects increase with film thickness. Thicker layers accumulate larger propagation phase and support stronger multiple internal reflections, which enhance both interference and helicity conversion. These results emphasize that quantitative modeling of VCD at oblique incidence requires the full 4 × 4 transfer-matrix treatment.

## Realistic Materials

To validate the transfer-matrix method against experimental data, we benchmark our simulations against the mid-IR VCD spectra of Mayerhöfer et al.,^35^ who presented experimentally extracted dielectric functions ε­(*ν̅*) and chirality admittances κ­(*ν̅*) for thin films of 1R-(+)-α-pinene and S-(−)-propylene oxide, together with the corresponding experimental VCD spectra. These optical constants were obtained from combined attenuated total reflection (ATR) and VCD measurements in the mid-infrared range and therefore provide a rigorous, parameter-free benchmark for our model.

Using the tabulated ε­(*ν̅*) and κ­(*ν̅*) from ref 35, we computed VCD spectra for plane-wave illumination at normal incidence, without any additional fitting parameters The resulting simulated VCD spectra are compared (Figure 7) with the experimental VCD curves reported by Mayerhöfer et al.^35^ for both 1R-(+)-α-pinene (*d* = 75 μm) and S-(−)-propylene oxide (*d* = 25 μm). Figure 7(a,b) shows that the TMM calculations (orange dashed lines) closely reproduce the experimentally measured spectra from ref 35 (solid blue lines). Such close agreement indicates that the TMM captures the intrinsic chiroptical response of molecular films without additional fitting parameters.

**Figure 7:**
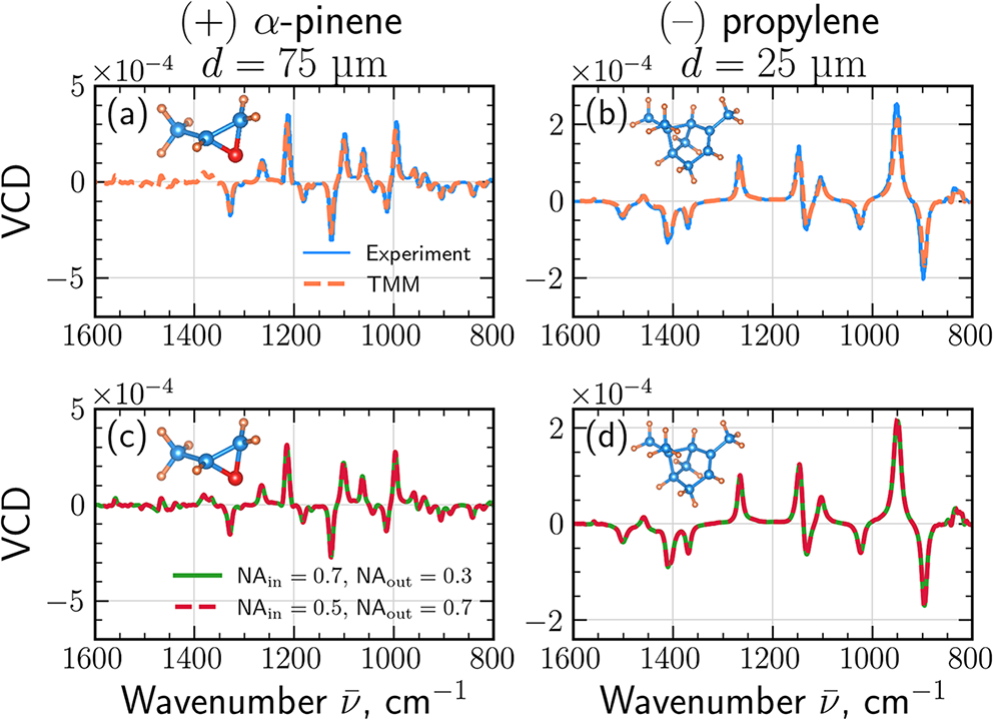
Comparison of TMM simulations with experimental VCD spectra reported by Mayerhöfer et al.^35^ and numerical aperture effects. (a) 1*R*-(+)-α-pinene film (*d* = 75 μm): experiment (solid blue lines) vs TMM calculation using the tabulated ε­(*ν̅*) and κ­(*ν̅*) (dashed red lines). (b) *S*-(−)-propylene oxide (*d* = 25 μm): experiment vs TMM. (c, d) VCD spectra obtained from the TMM model and averaged over two illumination/collection aperture pairs, (NA_in_, NA_out_) = (0.7, 0.3) (solid green lines) and (0.5, 0.7) (dashed red lines).

For a sample under focused illumination the aperture-averaged VCD spectrum is obtained by first averaging the transmittance for right-(*T*_+_) and left-(*T*_–_) circularly polarized light over all illumination and detection angles that fall within their respective numerical apertures

16 $${\mathrm{T̅}}_{\pm }\left(\mathrm{\nu ̅}\right)=\frac{1}{N_{\mathrm{in}}N_{\mathrm{out}}}\sum_{i=1}^{N_{\mathrm{in}}}\sum_{j=1}^{N_{\mathrm{out}}}T_{\pm }\left(\mathrm{\nu ̅},\theta_{i}^{\mathrm{in}},\theta_{j}^{\mathrm{out}}\right)$$

where *T*_±_ (*ν̅*, θ_*i*_^in^, θ_*j*_^out^) are the total helicity-resolved transmittances from eq 8, and the sums run over discrete angles θ_*i*_^in^ and θ_*j*_^out^ uniformly sampled within their respective NA ranges (with θ = arcsin­(NA)). In the symmetric configuration of air–sample–air layers, the refraction angles are identical on both sides, θ_*i*_^out^ = θ_*i*_^in^, so the averaging reduces to a single integral (or sum) over θ within the smaller of the two apertures.

Figure 7(c, d) examines the influence of finite numerical aperture by averaging the angle-dependent transmittances over representative aperture pairs (NA_in_, NA_out_) = (0.7, 0.3) and (0.5, 0.7), using Snell’s law of refraction for *n*_±_(*ν̅*). For these realistic materials, the spectra show only a weak dependence on NA. This behavior reflects the small magnitude of the chiral parameter (|κ| ≪ *n*) and the fact that κ­(*ν̅*) is generally out of phase with the absorption features of ε­(*ν̅*), producing nearly angle-independent phase retardation between the circular eigenmodes.

Taken together, the experimental agreement and minimal aperture sensitivity demonstrate that the transfer-matrix framework provides a robust and quantitative description of molecular VCD in the mid-infrared wavelength range.

## Discussion

Unlike mid-IR absorbance, VCD signals are exceptionally prone to artifacts, and significant work has been done to design and implement VCD systems to circumvent or overcome this problem when FT-IR systems were first designed. Early FT-IR VCD instruments and subsequent improvements were largely driven by the need to suppress optical artifacts through sophisticated polarization modulation, optical design, and sample preparation strategies.^3,9,11^ It is not only the system design but also the sample preparation that affects the observed VCD signal. There was no rigorous, physics-based model that would explain these artifacts, which remain a point of debate and discussion in the VCD community.

The present work deliberately focuses on a single homogeneous isotropic chiral layer to isolate and quantitatively dissect the two dominant electromagnetic mechanisms that distort the measured VCD signal beyond the intrinsic molecular circular dichroism: (i) thin-film interference and (ii) coupling between left- and right-circularly polarized (LCP and RCP) waves. The modular 4 × 4 transfer-matrix formalism naturally extends to multilayer stacks, substrates, supported films, and focused-beam geometries relevant to modern QCL-based VCD microscopy.^19,20,39^ Many real chiral samples exhibit additional structural complexity including optical anisotropy, surface roughness, spatial inhomogeneity, and mesoscopic organization, all of which can quantitatively modify the measured VCD response.

In isotropic molecular systems linear birefringence (LB) and linear dichroism (LD) are either absent in the steady-state linear response or appear only as weak, field-induced, or residual effects.^40^ Presented formulation allows isolation of the fundamental mechanisms of circular birefringence and circular dichroism without invoking tensorial dielectric functions. By adopting scalar permittivity and chirality parameters, we therefore capture the dominant sources of circular birefringence and circular dichroism under the standard assumptions of quantitative chiroptical spectroscopy.^9,41,42^ When LB/LD contributions do arise (e.g., due to partial molecular alignment, stress in solid films, or imperfect sample preparation), they are routinely suppressed experimentally in state-of-the-art mid-IR VCD setups through reflective high-NA optics, optimized modulation schemes, and dedicated correction protocols (quarter-wave plate compensation, Mueller matrix calibration, or postprocessing subtraction).^12,16,43,44^ Under these conditions, the residual linear artifacts are typically reduced by 1–2 orders of magnitude relative to the intrinsic chiral signal,^11^ so that the measured response is dominated by true circular effects. The angular averaging inherent to high-NA illumination and collection further mitigates any remaining angle-dependent linear contributions.

A central and somewhat counterintuitive finding of the present analysis is that tightly focused illumination (high numerical aperture) actually reduces geometric artifacts in mid-IR VCD rather than exacerbating them. Large oblique angles within the acceptance cone promote significant LCP–RCP polarization conversion and differential phase accumulation inside the film, which systematically diminishes the apparent VCD magnitude (see Figure 5).

While real high-NA refractive mid-IR optics can introduce chromatic aberration and wavelength-dependent focal shifts, these effects are well understood and quantitatively tractable. Importantly, these effects are not phenomenological in nature but can be quantitatively described by using established optical design frameworks. Standard ray-tracing and optical modeling tools (e.g., Zemax) allow accurate prediction of focal position and beam parameters as a function of wavelength, and such approaches have been successfully applied to discrete-frequency mid-IR absorbance measurements.^39^ This approach is particularly suitable for QCL-VCD measurements, which are performed sequentially at narrow, well-defined wavelengths, allowing focal shifts to be accounted for on a per-frequency basis.^19,20^ In the present work, no contributions of linear birefringence arising from the optical components were assumed, as the focus of this study is on other sources of baseline distortions. More complex optical systems in which additional linear birefringence may be introduced could be explored in future investigations.

The framework intentionally excludes setup-dependent nonidealities such as finite polarization purity, depolarization in the beam path, or misalignment, as these are highly instrument-specific and are usually minimized or corrected in practice. By concentrating on universal electromagnetic phenomena intrinsic to all mid-IR VCD measurements, the model establishes a transparent, quantitative baseline. Surface roughness and interfacial layers can be modeled by introducing one or more effective thin layers between the sample and the surrounding medium, with their optical response described using effective-medium theories (Maxwell Garnett approximation).^45–47^ Optical anisotropy can be incorporated within the present framework by extending the scalar material parameters to tensorial permittivity and chirality functions, ε­(*ν̅*) and κ­(*ν̅*), following the general 4 × 4 Berreman formalism.^31^ For samples exhibiting stronger spatial inhomogeneity or complex mesoscopic morphology beyond the validity of effective-medium approximations, the transfer-matrix approach can be naturally combined with rigorous numerical techniques, such as rigorous coupled-wave analysis (RCWA), finite-difference time-domain (FDTD), or finite-element methods (FEM), which provide full-wave solutions of Maxwell’s equations for arbitrarily structured chiral systems.

## Conclusions

In summary, we have presented a computational framework for modeling absorbance and vibrational circular dichroism in chiral thin films via the rigorous 4 × 4 transfer-matrix formalism. This approach allows us to examine systematically how film thickness, material losses, and the chiral parameter κ­(*ν̅*) contribute to both true VCD signals and optical artifacts. The transfer-matrix framework developed here bridges microscopic chiral oscillator models with macroscopic observables under realistic optical conditions, clarifies the physical origin of key artifacts, and establishes a rigorous theoretical basis for identifying conditions under which reliable mid-IR VCD spectra and imaging contrast can be obtained.

The main findings can be summarized as follows:

(i) In the achiral limit, thin films exhibit pronounced interference fringes that enhance the apparent absorption near the first resonance, whereas thicker layers approach the Beer–Lambert regime with nearly uniform peak intensities. This trend provides a clear baseline for identifying deviations caused by finite chirality.
(ii) For nonzero κ­(*ν̅*), the differential absorbance and resulting VCD signal emerge directly from the dispersive and absorptive parts of the chiral parameter. The magnitude of VCD scales with both κ and film thickness, establishing a transparent link between microscopic chirality and experimentally measurable dichroism.
(iii) Tightly focused illumination is advantageous for VCD measurements, as it minimizes the coupling between LCP and RCP waves and thus reduces optical artifacts.

## Data Availability

The data sets generated and analyzed during this study, including processed spectral data and analysis scripts, are available from the corresponding author upon reasonable request.
